# Effectiveness of upfront triple oral combination therapy with additional pirfenidone in a patient with severe pulmonary hypertension associated with lung diseases

**DOI:** 10.1002/rcr2.70010

**Published:** 2024-08-26

**Authors:** Fumihiro Kashizaki, Sachiko Matsumoto, Atsushi Miyasaka, Nanami Tsuchiya, Reeko Osada, Mai Kaneko, Kentaro Yumoto, Hao Chen, Kenji Konishi, Harumi Koizumi, Kenichi Takahashi, Takeshi Kaneko

**Affiliations:** ^1^ Department of Respiratory Medicine Yokohama Minami Kyosai Hospital Yokohama Japan; ^2^ Department of Respiratory Medicine Seirei Yokohama Hospital Yokohama Japan; ^3^ Department of Respiratory Medicine Yokohama City University Hospital Yokohama Japan

**Keywords:** chronic obstructive pulmonary disease, interstitial lung disease, pirfenidone, pulmonary hypertension, upfront triple combination therapy

## Abstract

Diagnosis and treatment of pulmonary hypertension (PH) in patients with lung diseases (PH‐LD) remain unestablished and pose significant challenges. In this report, we present a case of a 77‐year‐old patient with an indeterminate for usual interstitial pneumonia pattern along with chronic obstructive pulmonary disease, who developed groups 1 and 3 PH. Following diagnosis, upfront triple oral combination therapy (UTOCT) with macitentan, sildenafil, and selexipag was initiated. Stability in disease progression was achieved over 4 years with the addition of pirfenidone to address interstitial lung disease progression. To the best of our knowledge, this represents the first reported case of PH‐LD, where disease control was maintained with the addition of pirfenidone to UTOCT. This case suggests that some patients with PH‐LD, presenting with groups 1 and 3 PH, may benefit from combined UTOCT and antifibrotic agents, potentially improving symptoms and extending their prognosis.

## Graphical abstract

Pulmonary hypertension (PH) in patients with interstitial lung disease (ILD) (PH‐ILD) patients treated with upfront triple oral combination therapy (UTOCT) using macitentan, sildenafil, and selexipag, along with pirfenidone for fibrosis, achieved symptomatic relief for 4 years. This represents the first reported case of sustained disease control in PH‐ILD with pirfenidone added to UTOCT.

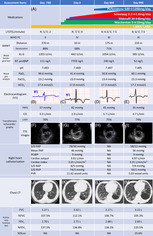

## INTRODUCTION

Patients with pulmonary hypertension (PH) associated with progressive interstitial lung disease (ILD) and chronic obstructive pulmonary disease (COPD) exhibit a prevalence of 30%–50%.^1^ These patients, classified as group 3 PH associated with lung diseases (PH‐LD),^2^ are less responsive to pulmonary vasodilators compared to those with pulmonary arterial hypertension (PAH). However, recent advancements suggest a mixed population involving elements of group 1, known as the PAH phenotype, such as systemic sclerosis‐associated severe ILD. Identifying PH‐LD patients with the PAH phenotype who may benefit from treatment is crucial owing to its poor prognosis.^3^, ^4^

In this report, we present a case where adding pirfenidone to upfront triple oral combination therapy (UTOCT) achieved symptom relief, potentially expanding treatment options for PH‐LD.

## CASE REPORT

**FIGURE 1:**
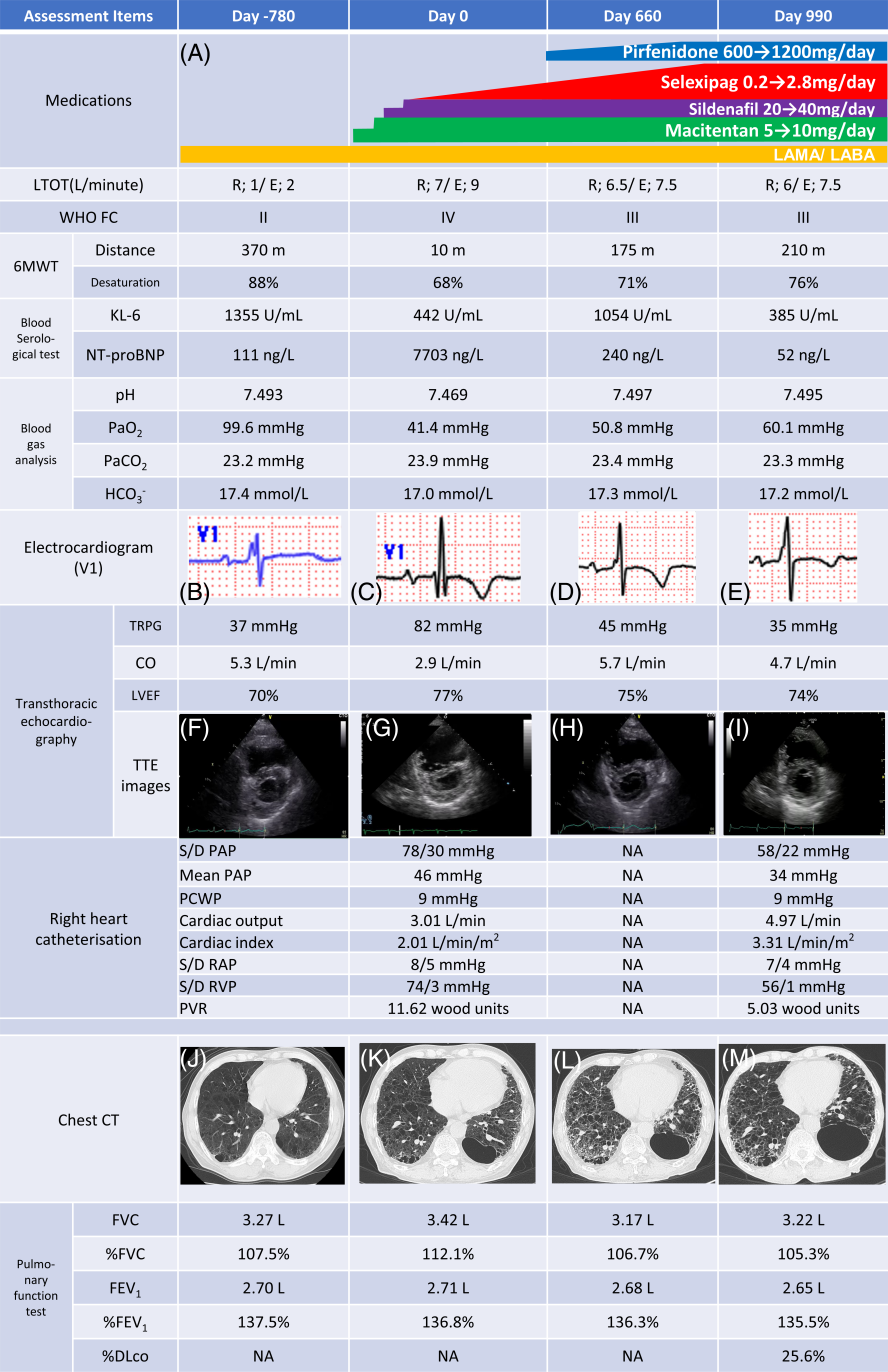
Clinical findings of the patient. (A) Clinical time course of drug administration. (B) An initial electrocardiogram (ECG) examination (Day 780) revealed an incomplete right bundle branch block in V1 induction. (C) The ECG on emergency admission (Day 0) displayed an increased R wave, negative T wave, and P wave alterations during V1 induction. (D) ECG findings during worsening interstitial pneumonia (Day 660) mirrored those observed in C. (E) ECG exhibited ameliorated T‐wave negativity compared with that on day D (Day 990). (F) Transthoracic echocardiography (TTE) of the parasternal left border short‐axis image at the initial visit (Day 780) revealed a peak tricuspid regurgitation velocity (TRV) of 2.2 m/s, tricuspid regurgitation peak gradient (TRPG) of 37 mmHg, cardiac output (CO) of 5.3 L/min, and no evidence of ventricular septal decompression. (G) Upon emergency admission (Day 0), the TTE of the parasternal left border short‐axis image displayed right ventricular enlargement, a D‐shaped image with left ventricular compression evident in both diastole and systole, a decrease in CO to 2.9 L/min, TRV of 4.5 m/s, and TRPG of 82 mmHg. (H) TTE performed at the time of worsening computed tomography (CT) findings of intra‐ and interlobular wall thickness and ground‐glass opacities (GGO) around the subpleural (Day 660) demonstrated a CO of 5.7 L/min, TRPG of 45 mmHg, and no flattening of the ventricular septal wall. (I) TTE revealed a CO of 4.7 L/min, a TRPG level of 35 mmHg, and no evidence of deterioration (Day 990). (J) Chest CT findings at the initial visit (Day 780) indicated no cardiac enlargement, relatively mild low‐attenuation areas (LAA), interlobular wall thickness, and GGO. The CT findings were predominantly LAA. (K) Upon emergency admission (Day 0), chest computed tomography revealed cardiac enlargement, worsening intra‐ and interlobular wall thickness, GGO around the subpleural, and dorsal cystic changes on the left side. These CT findings suggested a probable usual interstitial pneumonia (UIP) pattern and COPD. Pulmonary artery dilatation was also observed; however, the LAA remained unchanged. (L) During the gradual worsening of interstitial pneumonia, chest CT (Day 660) showed progressive intra‐ and interlobular wall thickness and widening of the GGO, although the cardiac enlargement improved. The left dorsal cyst was further enlarged, with no change in pulmonary artery dilation or LAA. Chest CT findings indicated a gradually more prominent probable UIP pattern. (M) Chest CT findings (Day 990) show improvement in GGO and intra‐ and interlobular wall thickness after the administration of pirfenidone. There was no cardiac enlargement; however, the left dorsal cyst was further enlarged, and the LAA became slightly prominent. The pulmonary artery dilation remained unchanged.

A 77‐year‐old former smoker with a 45‐pack‐year history was referred to our hospital for long‐term oxygen therapy (LTOT) because of COPD along with a computed tomography (CT) pattern of indeterminate for usual interstitial pneumonia. Chest CT revealed a predominantly central low attenuation area (LAA) and mild intra‐ and interlobar wall thickening, mainly in subpleural areas (Figure 1J). One month before his first visit, he experienced his second acute exacerbation of ILD and LTOT initiation. During his first visit, he received O_2_ supplementation at 1 L/min and a resting SpO_2_ of 94% (Figure 1, Day−780). Twenty‐six months later (Day 0), the patient presented to the emergency department with worsening dyspnoea. His vital signs included a temperature of 36.5°C, blood pressure of 99/70 mmHg, pulse of 95/min, respiratory rate of 26 breaths/min, and SpO_2_ of 78% (with O_2_ supplementation at 1 L/min) (Figure 1, Day 0). His World Health Organization functional class (WHO FC) deteriorated from II to IV. During the physical examination, digital clubbing, fine crackles in the lung fields, increased IIp heart sounds, and 1+ oedema in the lower extremities were noted. Haematological findings included a white blood cell count of 7810/μL, lactate dehydrogenase 233 IU/L, blood urea nitrogen 26.0 mg/dL, creatinine 1.04 mg/dL, c‐reactive protein 0.17 mg/dL, KL‐6442 U/mL, and NT‐proBNP 7703 ng/L. Autoantibodies tested negative. Chest CT showed that the left lower cyst had enlarged, with progressive intra‐ and interlobar wall thickening and ground‐glass opacities (GGO) around the subpleural lesions, marked cardiac enlargement, and pulmonary artery dilation (Figure 1K). Right heart catheterisation (RHC) revealed either group 1, group 3, or both (Figure 1, Day 0). Furosemide (40 mg/day) was initiated but failed to achieve improvement. At the patient's request, macitentan was initiated at 5 mg/day and increased to 10 mg/day. Although effective, the response was inadequate, so other vasodilators were added. Sildenafil was increased to 40 mg/day, and selexipag was added at 0.2 mg/day. The patient was transitioned to triple therapy over approximately 4 weeks (Figure 1A). His symptoms and respiratory condition improved with no adverse events except mild nasal discharge and headache due to sildenafil following the introduction of UTOCT. Pirfenidone was initiated at 600 mg/day, later increased to 1200 mg/day owing to elevated KL‐6 levels, progression of intra‐ and interlobar wall thickening, GGO on CT, and dyspnoea on exertion (Figure 1L). RHC findings revealed improvement compared to pre‐UTOCT treatment. Despite continuous treatment for 4 years, worsening dyspnoea due to the deterioration of PH‐LD ultimately required primarily palliative care.

## DISCUSSION

In this report, UTOCT was administered to assess treatment efficacy and safety, with the addition of pirfenidone to address CT findings of intra‐ and interlobar wall thickening, GGO, and progression of dyspnoea on exertion, maintaining symptomatic relief for approximately 4 years.

The pathophysiology of PH in COPD and ILD is traditionally attributed to hypoxic pulmonary vasoconstriction and the loss of the pulmonary vascular bed due to lung destruction.^3^ However, airway inflammation can also cause vascular endothelial cell damage, leading to abnormal proliferation of pulmonary artery endothelial and smooth muscle cells.^3^ Smoking has been reported to cause vascular endothelial cell damage and increased pulmonary arterial pressure in animal studies.^3^ Notably, plexiform lesions were observed in the pathology of resected lungs during lung transplantation, regardless of ILD progression.^5^

Patients with PH‐LD have a poorer prognosis than those with PAH without lung disease. Various pulmonary vasodilators have been studied for PH‐LD, with inhaled treprostinil demonstrating efficacy and becoming a central treatment for some patients.^4^ In cases where treatment effects are inadequate or adverse events occur, switching to or adding additional drugs may improve outcomes. Upfront triple combination therapy is standard for PAH,^3^ and switching from inhaled treprostinil to oral selexipag may offer similar safety and efficacy,^6^ suggesting benefits for some PH‐LD patients.

In this case, CT findings of intra‐ and interlobar wall thickening and GGO, as well as serum KL‐6 levels, deteriorated despite UTOCT administration. The addition of pirfenidone mitigated the progression of these findings. Recent studies report that vascular endothelial growth factor (VEGF) receptor inhibitors induce PH through endothelial cell proliferation in a PAH rat model.^7^ Furthermore, sotatercept, which targets TGF‐β pathway inactivation, improves right ventricular function after 24 weeks of treatment.^8^ Although its mechanism is not fully understood, pirfenidone suppresses TGF‐β production without inhibiting VEGF. Therefore, adding pirfenidone to UTOCT may provide a synergistic antifibrotic effect in some PH‐LD patients.

To the best of our knowledge, this is the first reported case of PH‐LD with disease control maintained for 4 years by adding pirfenidone to UTOCT. Some respiratory disease patients whose hypoxemia is controlled with LTOT may have PH responsive to pulmonary vasodilators. Further research is needed to improve the prognosis of PH‐LD patients.

## AUTHOR CONTRIBUTIONS

Fumihiro Kashizaki interpreted the data and drafted the original and revised manuscript. Hao Chen amd Kenji Konishi significantly contributed to data analysis and interpretation. Sachiko Matsumoto, Atsushi Miyasaka, Nanami Tsuchiya, Reeko Osada, Mai Kaneko, and Kentaro Yumoto were responsible for data curation. Harumi Koizumi, Kenichi Takahashi, and Takeshi Kankeko made substantial contributions to revising the manuscript drafts. All authors have reviewed and approved the final version of the manuscript and agree to be accountable for their respective contributions to the work.

## CONFLICT OF INTEREST STATEMENT

None declared.

## ETHICS STATEMENT

The authors declare that appropriate written informed consent was obtained for the publication of this manuscript and accompanying images.

## Data Availability

The data that support the findings of this study are available on request from the corresponding author. The data are not publicly available due to privacy or ethical restrictions.

## References

[1] Cottin V , Le Pavec J , Prévot G , Mal H , Humbert M , Simonneau G , et al. Pulmonary hypertension in patients with combined pulmonary fibrosis and emphysema syndrome. Eur Respir J. 2010;35:105–111. 19643948 10.1183/09031936.00038709

[2] Humbert M , Kovacs G , Hoeper MM , Badagliacca R , Berger RMF , Brida M , et al. 2022 ESC/ERS guidelines for the diagnosis and treatment of pulmonary hypertension. Eur Heart J. 2022;43:3618–3731. 36017548 10.1093/eurheartj/ehac237

[3] Piccari L , Allwood B , Antoniou K , Chung JH , Hassoun PM , Nikkho SM , et al. Pathogenesis, clinical features, and phenotypes of pulmonary hypertension associated with interstitial lung disease: a consensus statement from the pulmonary vascular research Institute's innovative drug development initiative ‐ group 3 pulmonary hypertension. Pulm Circ. 2023;13:e12213. 37025209 10.1002/pul2.12213 PMC10071306

[4] Nathan SD , Johri S , Joly JM , King CS , Raina A , McEvoy CA , et al. Survival analysis from the INCREASE study in PH‐ILD: evaluating the impact of treatment crossover on overall mortality. Thorax. 2024;79:301–306. 37979971 10.1136/thorax-2023-220821 PMC10958253

[5] Dotan Y , Stewart J , Gangemi A , Wang H , Aneja A , Chakraborty B , et al. Pulmonary vasculopathy in explanted lungs from patients with interstitial lung disease undergoing lung transplantation. BMJ Open Respir Res. 2020;7:e000532. 10.1136/bmjresp-2019-000532 PMC7359183 32661103

[6] Frost A , Janmohamed M , Fritz JS , McConnell JW , Poch D , Fortin TA , et al. Safety and tolerability of transition from inhaled treprostinil to oral selexipag in pulmonary arterial hypertension: results from the TRANSIT‐1 study. J Heart Lung Transplant. 2019;38:43–50. 30391194 10.1016/j.healun.2018.09.003

[7] Taraseviciene‐Stewart L , Kasahara Y , Alger L , Hirth P , Mc Mahon G , Waltenberger J , et al. Inhibition of the VEGF receptor 2 combined with chronic hypoxia causes cell death‐dependent pulmonary endothelial cell proliferation and severe pulmonary hypertension. FASEB J. 2001;15:427–438. 11156958 10.1096/fj.00-0343com

[8] Hoeper MM , Badesch DB , Ghofrani HA , Gibbs JSR , Gomberg‐Maitland M , McLaughlin VV , et al. Phase 3 trial of sotatercept for treatment of pulmonary arterial hypertension. N Engl J Med. 2023;388:1478–1490. 36877098 10.1056/NEJMoa2213558

